# Successful Percutaneous Closure of Gerbode Defect and Right Atrial-Aortic Fistula Following Infective Endocarditis

**DOI:** 10.1016/j.jaccas.2024.102410

**Published:** 2024-06-20

**Authors:** Muhammad Samsoor Zarak, Sulaiman Rathore, Raj K. Bose, Rajesh Janardhanan, Dexter DeLeon

**Affiliations:** a Department of Internal Medicine, Northwest Medical Center, Tucson, Arizona, USA; b Division of Cardiology, Northwest Medical Center, Tucson, Arizona, USA; c PIMA Heart and Vascular, Tucson, Arizona, USA; d Sarver Heart Center, Banner University Medical Center, Tucson, Arizona, USA

**Keywords:** atrial-aortic fistula, Gerbode defect, infective endocarditis, percutaneous approach, septal defects

## Abstract

We report a case of infective endocarditis with a septal abscess that was complicated with abnormal blood flow from the left ventricle to the right atrium (Gerbode defect) along with abnormal blood flow from the aorta to the right atrium (atrial-aortic fistula). This is the first reported case of successful correction of both defects by a percutaneous approach.

## Graphical Abstract

**Figure:**
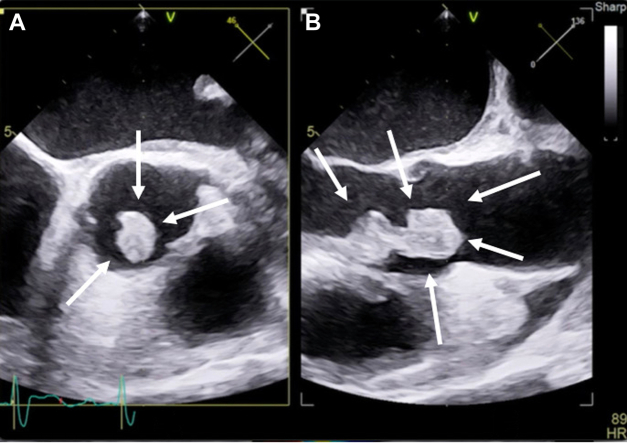

## History of Presentation

The patient was a 63-year-old man who presented with unexplained fatigue, unintentional weight loss of 20 lbs, and a lower extremity nonpurpuric rash. He was afebrile, with blood pressure of 123/47 mm Hg, a heart rate of 81 beats/min, and a respiratory rate of 18 breaths/min. On examination, the patient had numerous palpable nonpruritic rash lesions on the bilateral lower extremities (left more than right) with sparing of the soles, his lungs were clear on auscultation, he had a regular heart rate without any murmur, no jugular venous pressure elevation was present, and he had no neurologic deficits. A complete blood count showed normal white blood cells, red blood cells, and platelets. Other laboratory tests showed an elevated creatinine level of 1.50 mg/dL, and urinalysis results were unremarkable. The patient was admitted to the medical service for management of acute kidney injury. On day 2, he developed high-grade fevers.

**Learning Objectives**

• To recognize the clinical presentation, diagnostic approach, and management strategies for infective endocarditis complicated by a Gerbode defect and an RAAF.
• To understand the decision-making process and outcomes of transcatheter vs surgical interventions in high-risk cardiac patients.

## Past Medical History

The patient had no known medical conditions, except that he had presented to the emergency department 2 weeks earlier with symptoms of a urinary tract infection and was empirically treated with cefalexin.

## Differential Diagnosis

Considering the lower extremity rash, a cephalexin-related skin reaction was the top differential diagnosis because cephalexin was the only medication the patient kept taking. However, his fatigue and weight loss were unexplained.

## Investigations

With the episode of high-grade fevers, blood cultures were obtained, and the results revealed gram-positive cocci in chains that eventually were identified as *Streptococcus anginosus* group. A transthoracic echocardiogram revealed a large vegetation on the aortic valve.

## Management

**Figure 1:**
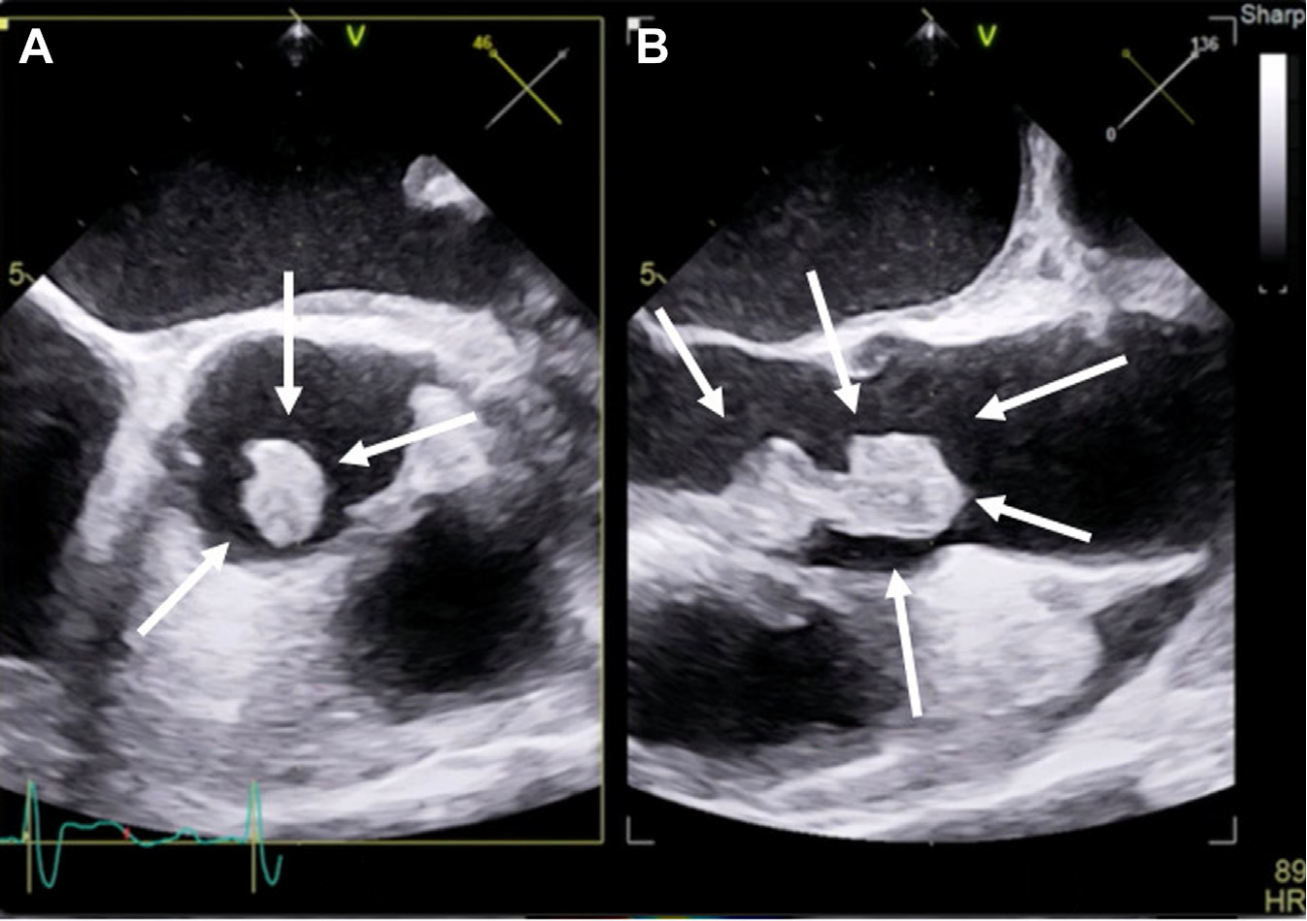
Transesophageal Echocardiographic Biplane Image at the Aortic Valve Level (A) Short-axis view. (B) Long-axis view. Images show a large vegetation attached to the right coronary cusp of aortic valve prolapsing to left ventricular outflow tract (arrows). There is coaptation of aortic valve leaflets.

**Figure 2:**
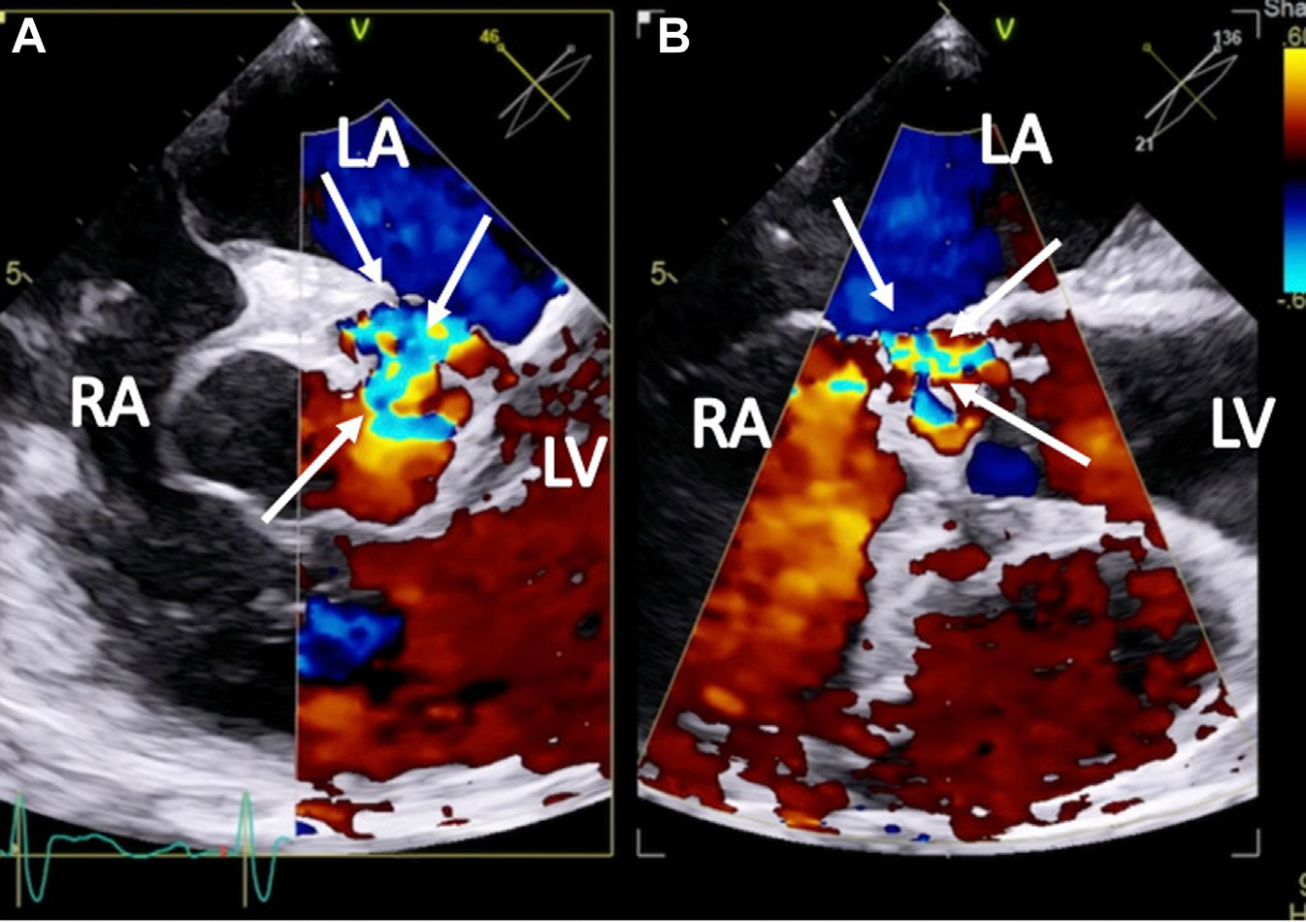
Transesophageal Echocardiographic Biplane Image at the Aortic Valve Level With Color Flow Doppler During Diastole (A) Short-axis view. (B) Long-axis view. The arrows demonstrate severe, anteriorly directed aortic regurgitation. LA = left atrium; LV = left ventricle; RA = right atrium.

Intravenous (IV) vancomycin was started after receipt of positive culture results, and an infectious disease specialist was consulted. The patient did not show response to antibiotic therapy and remained febrile the following day. He eventually became confused, and that raised concern for a septic cerebral embolism. The patient was transferred to the intensive care unit. Cardiac magnetic resonance (CMR) showed punctate septic emboli to the left splenium of the corpus callosum, the right periventricular centrum semiovale, and the right subcortical right frontal lobe. The patient’s hospital course was further complicated by bradycardia, which eventually progressed to complete heart block requiring placement of a temporary pacemaker. A transesophageal echocardiogram (TEE) revealed a large vegetation attached to the aortic valve and prolapsing into the LV outflow tract with evidence of severe anteriorly directed aortic regurgitation and possible communication between the aortic root and the right ventricular (RV) septum (Figures 1A and 1B and 2A and 2B, Videos 1 and 2). Cardiothoracic surgery was performed for aortic valve replacement and right atrial, RV, and aortic annular repair using a CorMatrix patch (CorMatrix Cardiovascular). Postoperatively, a dual-chamber CMR-compatible pacemaker was inserted. After 12 days, the patient’s condition improved, and he was eventually discharged to a rehabilitation facility to complete 6 weeks of IV ceftriaxone (2 g).

**Figure 3:**
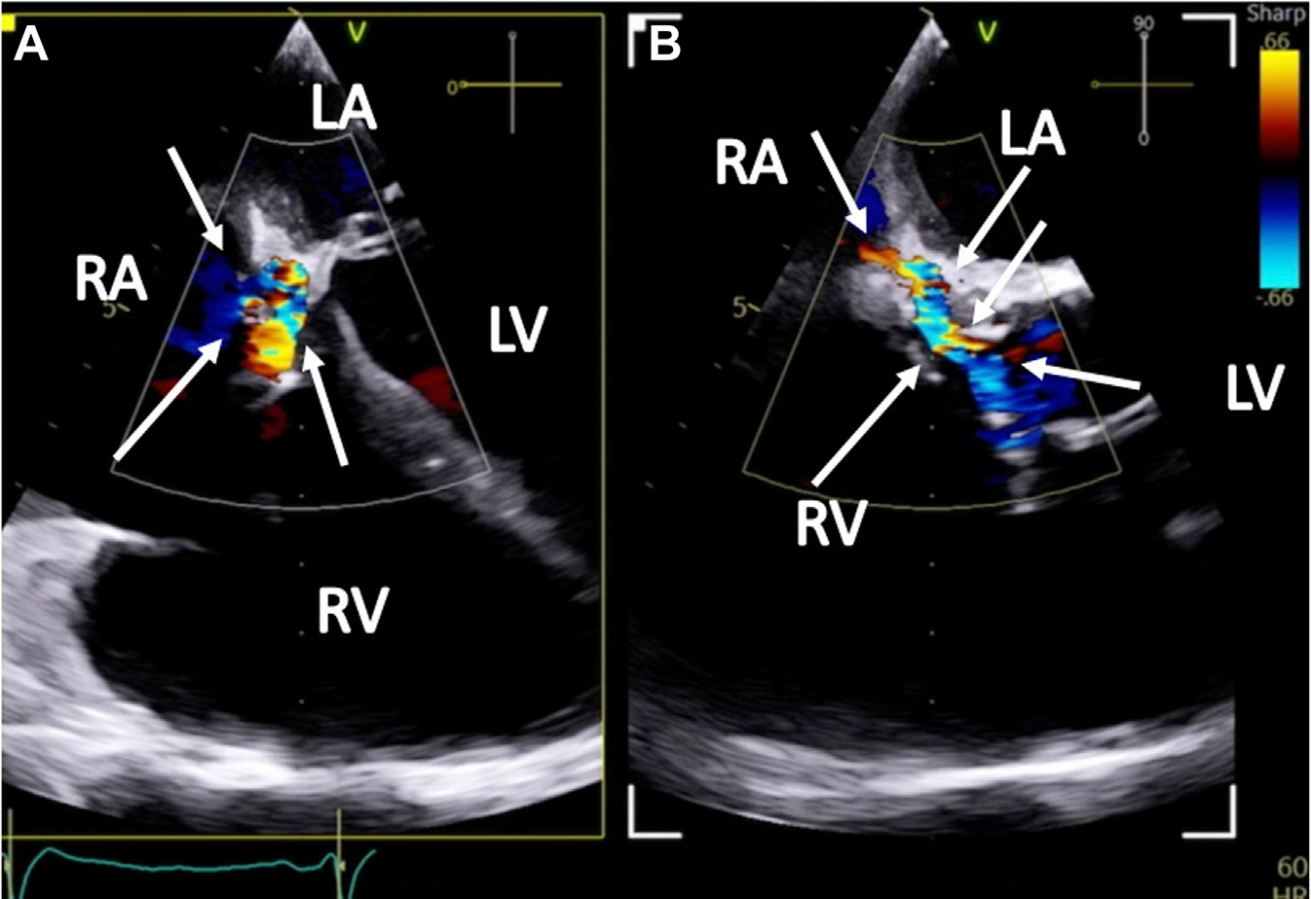
Transesophageal Echocardiographic Biplane Images at the Midesophageal Level With Color Flow Doppler (A and B) The arrows demonstrate abnormal flow between the aorta and the right atrium (RV; fistula between the aorta and the right atrium). Abbreviations as in Figure 2.

**Figure 4:**
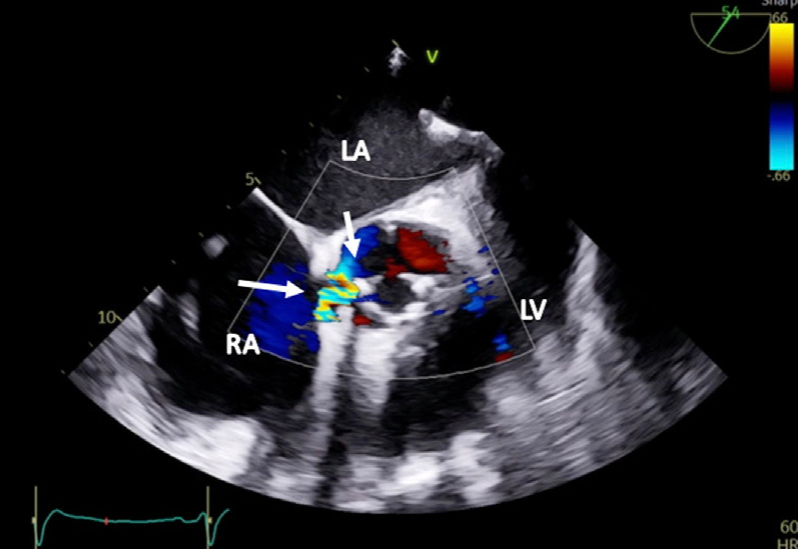
Transesophageal Echocardiographic Image at the Midesophageal Long Axis With Color Flow Doppler Demonstrating Flow Between Right Coronary Cusp to the RA The arrow demonstrates abnormal flow between right coronary cusp to right atrium. Abbreviations as in Figure 2.

**Figure 5:**
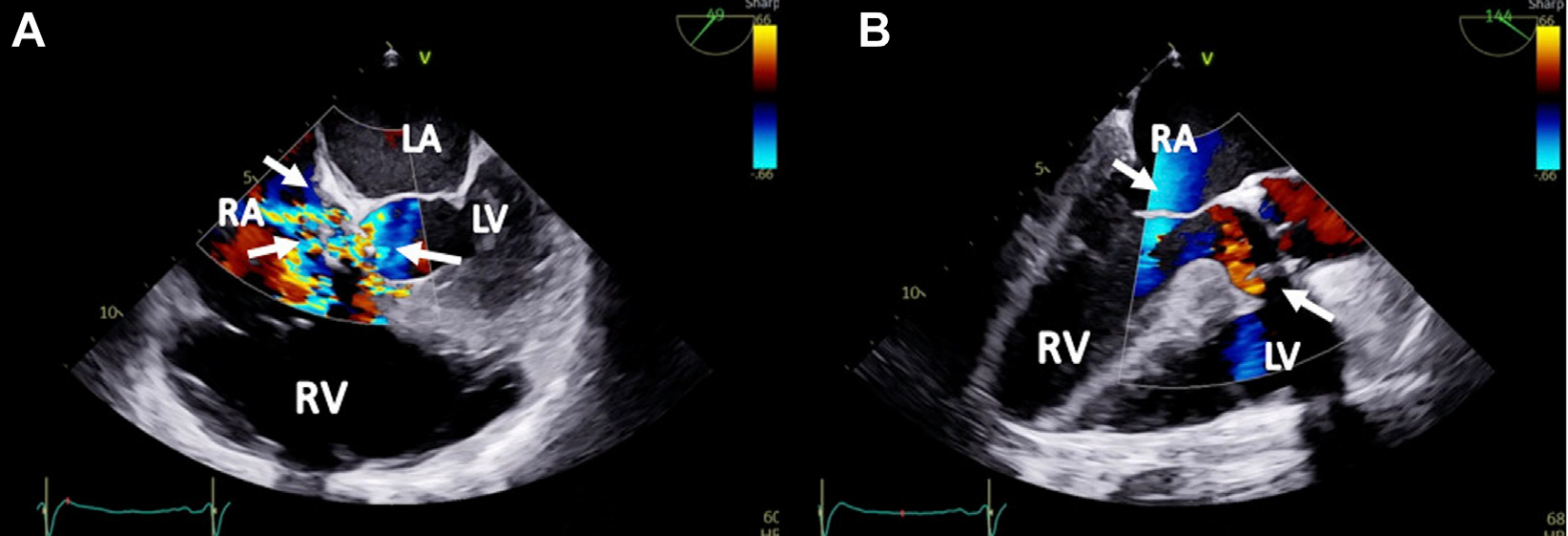
Transesophageal Echocardiographic images (A) Midesophageal 4-chamber view showing a Gerbode defect with color flow between the left ventricle (LV) and the right atrium (RA), with systolic color flow. (B) Long-axis view with color flow Doppler showing abnormal flow between the left ventricle and the right atrium. Abbreviations as in Figures 2 and 3.

**Figure 6:**
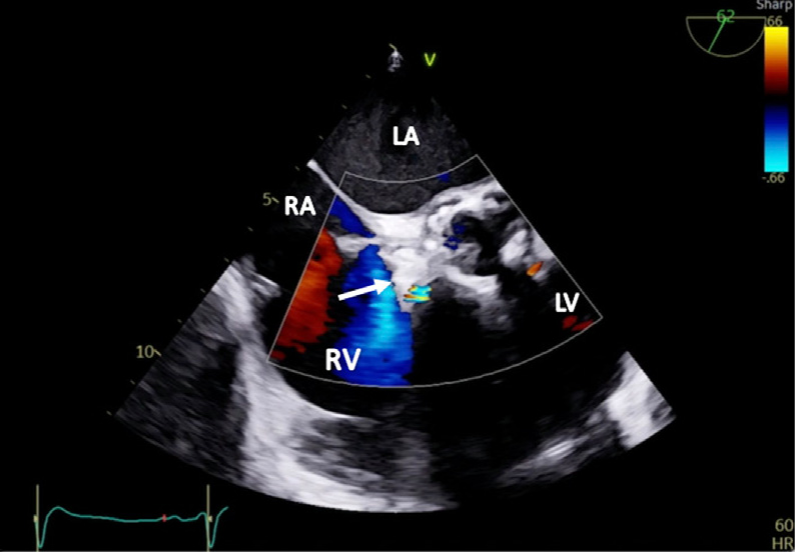
Transesophageal Echocardiographic Short-Axis View at the Aortic Valve Level With Color Flow Doppler Showing Successful Closure of the Right Atrial-Aortic Fistula The arrow shows an occluder device. Abbreviations as in Figures 2 and 3.

**Figure 7:**
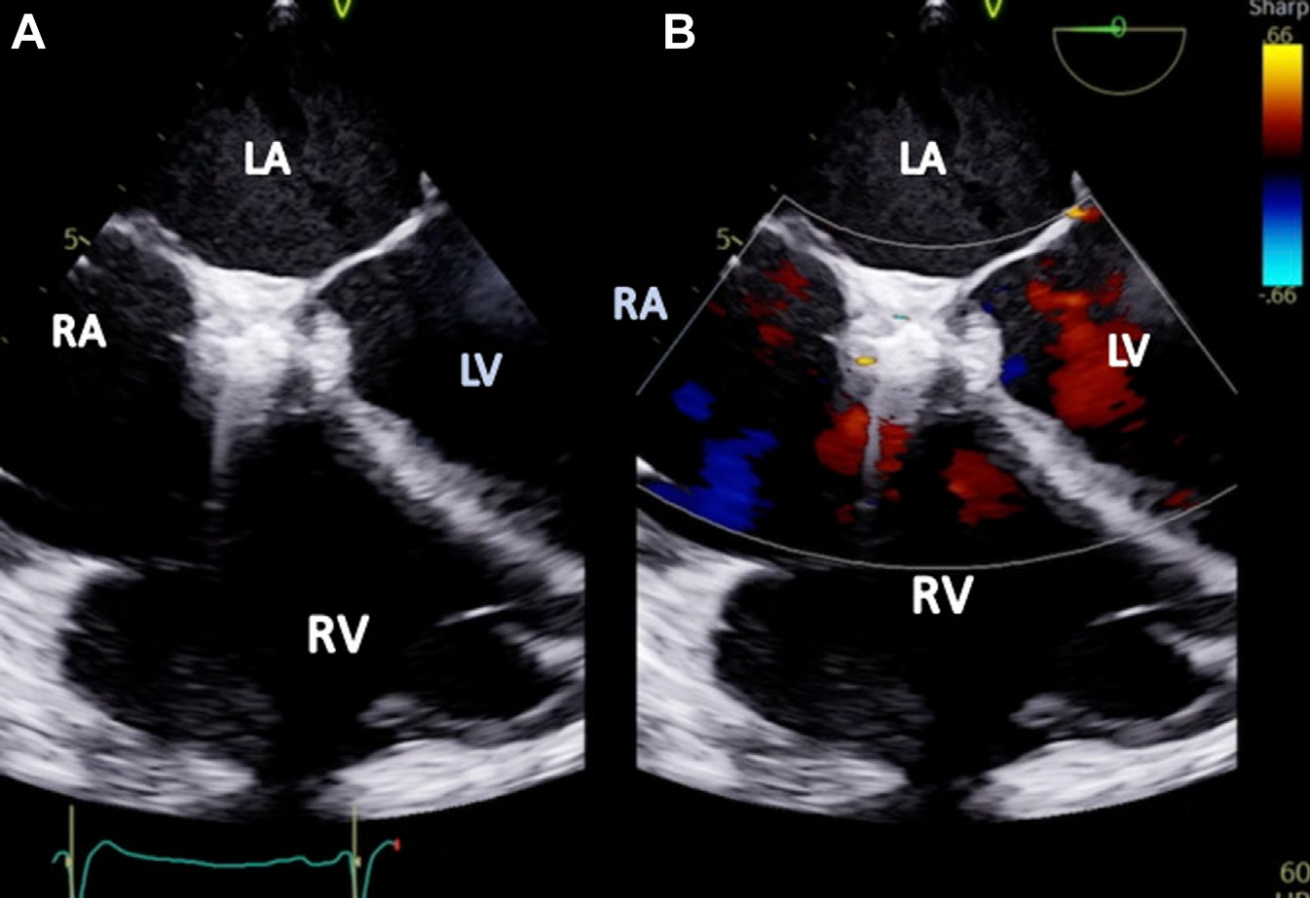
Transesophageal Echocardiographic Images Showing Gerbode Defect Closure (A) Without Doppler. (B) With color Doppler. These images did not reveal any flow between the left ventricle (LV) and the right atrium (RA), and they show successful closure of the Gerbode defect. Abbreviations as in Figures 2 and 3.

Two weeks later, the patient was readmitted with acute heart failure and was found to have a new onset murmur, anasarca, and cardiogenic shock. The patient did not respond well to guideline-directed medical therapy (GDMT), and he was eventually started on continuous renal replacement therapy. A TEE showed abnormal flow from the right coronary cusp into the right atrium (Figures 3A and 3B and 4, Videos 3 and 4). A TEE and an angiogram also confirmed a 5-mm fistula from the right coronary sinus of Valsalva into the right atrium and a 10-mm ventricular septal defect (VSD) to the right atrium with a pseudoaneurysm (Figures 5A and 5B, Video 5, Video 6, Video 7). The heart team deemed the patient a prohibitive risk for redo open cardiac surgery, and percutaneous defect closure was then proposed. Femoral venous and arterial access was obtained. The right atrial-aortic fistula (RAAF) was first crossed through a retrograde approach using a 0.018-inch wire. The wire was externalized, and a 0.035-inch wire was buddied as a bail-out wire. A Judkins right (JR)4 guide was inserted through the right femoral vein over the 0.018-inch wire, and another wire was then inserted and used to attach the right atrium to the VSD. The ventricular wire was then delivered across the aortic valve, and a Confida 0.035-inch wire (Medtronic) was inserted into the left ventricle to accommodate a VSD closure device delivery system. A 14-mm Amplatzer VSD closure device (Abbott) was deployed. A 10 × 8 Amplatzer patent ductus arteriosus occluder was delivered to the RAAF. Aortography and TEE confirmed the proper placement of the closure devices without any interaction with the aortic, mitral, and tricuspid valves (Videos 8 and 9). The devices were released after significant flow resolution was seen on color flow Doppler imaging. After the procedure, the hemodynamics significantly improved, and closure was confirmed with TEE (Figures 6 and 7A and 7B, Videos 10 and 11). The patient improved gradually. He started making urine and was tolerating GDMT. He was discharged to a rehabilitation facility 15 days after fistula closure. On follow-up after 2 weeks, the patient remained hemodynamically stable and had NYHA functional class I /II symptoms with GDMT.

## Discussion

Our patient presented with infective endocarditis complicated with an aortic and interventricular septal abscess, resulting in an acquired Gerbode defect and RAAF. Both defects led to heart failure that did not respond to medical therapy. Given his acute heart and multiorgan failure, he was deemed prohibitive for redo open surgical closure; the patient underwent a successful corrective measure as a bridge to surgery through a transcatheter approach. To our knowledge, this is the first reported case of simultaneous percutaneous closure of both defects.

The Gerbode defect is a VSD connecting the left ventricle and right atrium. The defect may be congenital or can happen after any cardiac insult, including myocardial infarction in the right coronary artery distribution, infective endocarditis, and blunt cardiac trauma or after cardiac surgery injuring the atrioventricular septum and creating an abnormal shunting of blood.^1^^,^^2^ An untreated Gerbode defect leads to congestive heart failure, valvular leaflet perforation, and complete heart block.^3^^,^^4^ Management depends on the severity of the case, and surgical closure is primarily recommended.^5^^,^^6^ Atrial-aortic fistula (AAF) is an abnormal pathologic connection that can also be congenital or acquired. AAF is commonly found secondary to other pathologic conditions, which include endocarditis, previous cardiac surgery, aortic dissection or aneurysm, ruptured sinus of Valsalva aneurysm, iatrogenic conditions, or vasculitis.^3^ Complications of AAF include congestive heart failure. Because this disease is very rare, therefore, there is limited information available in the literature.^7^

Given that RAAF is rare, limited evidence is available for its correction through the transcatheter approach;^3^ however, there is a published report in which corrective measures were obtained by a surgical approach.^8^^,^^9^ For the Gerbode defect, the literature indicates a preference for surgical closure, thus providing a definitive solution by restoring normal cardiac anatomy. However, this approach poses various risks, including an operative risk, cardiac complications, pulmonary issues, postoperative bleeding, and thromboembolism, especially for a patient in shock and with fulminant heart and multiorgan failure.^10^

In contrast, scarce literature supports the transcatheter approach for Gerbode defect management.^3^ Nevertheless, the advantages of the transcatheter approach lie in being minimally invasive, avoiding sternotomy, enabling quick recovery, causing less impact on surrounding tissues, and resulting in a comparatively shorter hospital stay.^11^ Potential drawbacks include incomplete closure, new infections, device-related complications, and arrhythmias.^12^^,^^13^

The choice between transcatheter and surgical management hinges on factors such as concomitant comorbid conditions and surgeon and patient preferences. In our high-risk patient with concomitant defects, where redo surgery was not recommended, we successfully closed both defects by using a transcatheter approach.

## Follow-Up

The patient was seen in the clinic 2 weeks after discharge. At that time, his status was NYHA functional class I. The patient’s condition was compensated with GDMT.

## Conclusions

Our case highlights the successful use of a transcatheter approach for a high-risk patient with a concomitant Gerbode defect and RAAF. Despite the traditional preference for surgical closure, our experience underscores the viability of individualized transcatheter strategies in complex cardiac scenarios. This case contributes to the evolving landscape of cardiac interventions and emphasizes the need for personalized patient care and ongoing research to refine transcatheter applications in diverse clinical contexts.

## Funding Support and Author Disclosures

The authors have reported that they have no relationships relevant to the contents of this paper to disclose.

## Appendix

Video 1 TEE Biplane View at the Aortic Valve Level Short axis on A, and Long Axis on B. Video show a large vegetation attached to the right coronary cusp of aortic valve prolapsing to LVOT. The coaptation of aortic valve leaflets is also noted.

Video 2 Biplane TEE View at the Aortic Valve Level With Color Flow Doppler During Diastole Short axis on A, and Long Axis on B. The video demonstrates severe anteriorly directed Aortic regurgitation.

Video 3 TEE Biplane View at the Midesophageal Level With Color Flow Doppler The video demonstrate abnormal flow between aorta and right atrium (fistula between aorta and right atrium).

Video 4 TEE at the Midesophageal Long-Axis With Color Flow Doppler Demonstrating Flow Between the Right Coronary Cusp and the Right Atrium

Video 5 TEE at the Midesophageal 4-Chamber View Demonstrating a Gerbode Defect With Color Flow Between the Left Ventricle and the Right Atrium With Systolic Color Flow

Video 6 TEE Long-Axis View With Color Flow Doppler Showing Abnormal Flow Between the Left Ventricle and the Right Atrium

Video 7 Angiogram Showing Flow Between the Left Ventricle and the Right Atrium Suggestive of a Gerbode Defect

Video 8 Angiogram Showing the Placement of a PDA Closure Device and a VSD Closure Device

Video 9 Angiogram Showing the Correct Placement of the PDA and VSD Closure Devices Mild flow is seen around the VSD device.

Video 10 TEE Short-Axis View at the Aortic Valve Level With Color Flow Doppler Showing Successful Closure of a Right Atrial-Aortic Fistula With a PDA Closure Device

Video 11 TEE Biplane View With and Without Color Doppler Does Not Reveal Any Flow Between the Left Ventricle and the Right Atrium Transesophageal echocardiography shows successful closure of the Gerbode defect.

## References

[1] Gerbode F., Hultgren H., Melrose D., Osborn J. (1958). Syndrome of left ventricular-right atrial shunt: successful surgical repair of defect in five cases, with observation of bradycardia on closure. Ann Surg.

[2] Silbiger J.J., Kamran M., Handwerker S., Kumar N., Marcali M. (2009). The Gerbode defect: left ventricular to right atrial communication—anatomic, hemodynamic, and echocardiographic features. Echocardiography.

[3] Amat-Santos I.J., Rojas P., Stella P.R. (2018). Intracardiac shunts following transcatheter aortic valve implantation: a multicentre study. EuroIntervention.

[4] Saker E., Bahri G.N., Montalbano M.J. (2017). Gerbode defect: a comprehensive review of its history, anatomy, embryology, pathophysiology, diagnosis, and treatment. J Saudi Heart Assoc.

[5] Kelle A.M., Young L., Kaushal S., Duffy C.E., Anderson R.H., Backer C.L. (2009). The Gerbode defect: the significance of a left ventricular to right atrial shunt. Cardiol Young.

[6] Beliaev A.M. (2020). How to repair an acquired Gerbode defect using an aortic root xenograft. J Card Surg.

[7] Jainandunsing J.S., Linnemann R., Maessen J. (2019). Aorto-atrial fistula formation and therapy. J Thorac Dis.

[8] Fierro E.A., Sikachi R.R., Agrawal A., Verma I., Ojrzanowski M., Sahni S. (2018). Aorto-atrial fistulas. Cardiol Rev.

[9] Ahmad T., Chithiraichelvan S., Patil T.A., Jawali V. (2014). Aortic root to left-atrial fistula after aortic valve replacement: a rare complication and its intraoperative management. Ann Card Anaesth.

[10] Uchida T., Kuroda Y., Kobayashi K., Sadahiro M. (2020). Surgical treatment for left ventricular–aortic discontinuity and Gerbode defect with endocarditis. Interact Cardiovasc Thorac Surg.

[11] Fanari Z., Barekatain A., Abraham N., Hopkins J.T. (2015). Percutaneous closure of acquired Gerbode defect: management of a rare complication of cardiac surgery. Interact Cardiovasc Thorac Surg.

[12] Sperlongano S., Scognamiglio G., D’Andrea A., Golino P. (2019). A systolic murmur late after infective endocarditis: looking for the guilty. J Cardiovasc Echogr.

[13] Sinisalo J., Sreeram N., Qureshi S.A. (2013). Transcatheter closure of acquired left ventricle to right atrium shunts. Catheter Cardiovasc Interv.

